# Supplementary material for: An extended interval between vaccination and infection enhances hybrid immunity against SARS-CoV-2 variants
Source: JCI Insight. 2023 Mar 8;8(5):e165265. doi: 10.1172/jci.insight.165265 (PMC10077480; doi:10.1172/jci.insight.165265)
Supplement: Supplemental data [file jciinsight-8-165265-s149.pdf]

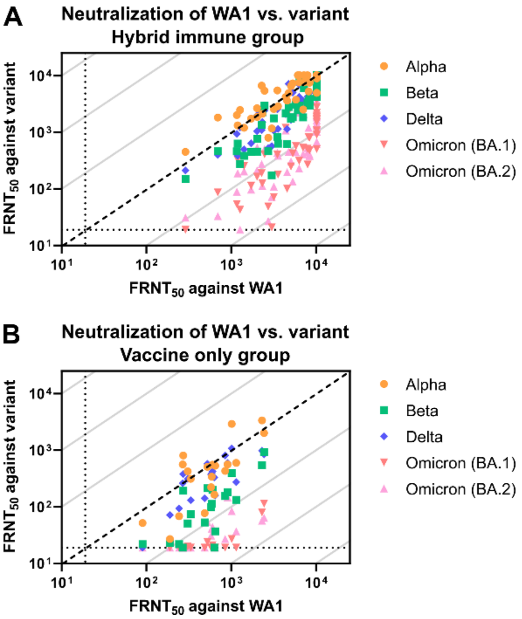

Supplemental figure 1: Variant cross-neutralization by hybrid immune sera is improved compared to vaccination alone

Individual neutralizing $FRNT_{50}$ values for each of the variants against WA1 for the hybrid immune group (**A**), and two-dose vaccine only group (**B**). Diagonal broken line indicates equal neutralization of WA1 and variant. For panels A-B, n=20 for the vaccine only group and n=46 for the hybrid immunity group.

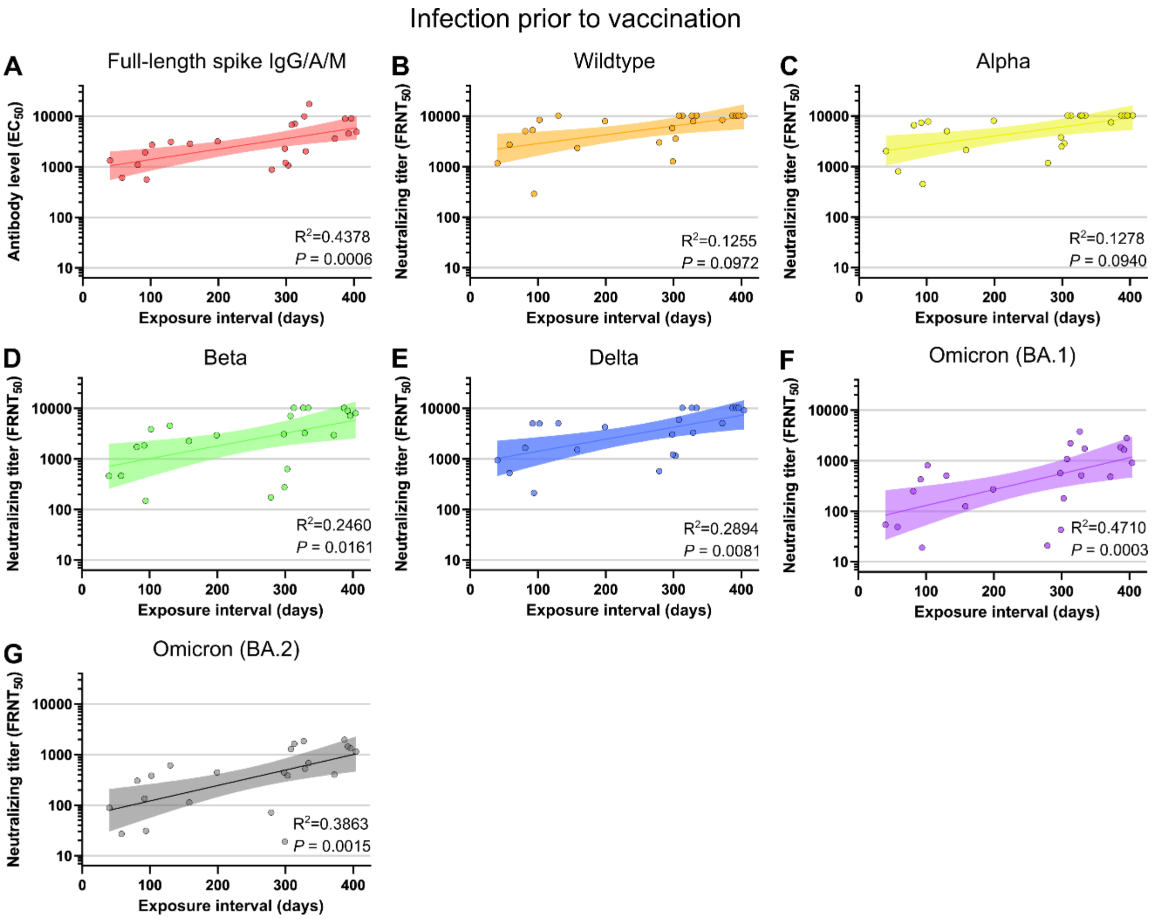

Supplemental figure 2: Infection prior to vaccination group neutralizing responses correlate with exposure interval

Comparison of exposure interval, the time between first and last antigen exposure, among individuals with SARS-CoV-2 infection prior to vaccination. Correlations are shown for full-length spike EC_{50} antibody levels (**A**), and neutralization of WA1 (**B**), Alpha (**C**), Beta (**D**), Delta (**E**), Omicron (BA.1) (**F**), and Omicron (BA.2) (**G**). Individual values are shown as filled circles and the shaded area indicates the linear fit with 95% confidence interval. R^2 is indicated for each curve fit and P values show the result of an F-test using a zero slope null hypothesis. All P values are two-tailed and 0.05 was considered significant. For panels A-G, n=23.

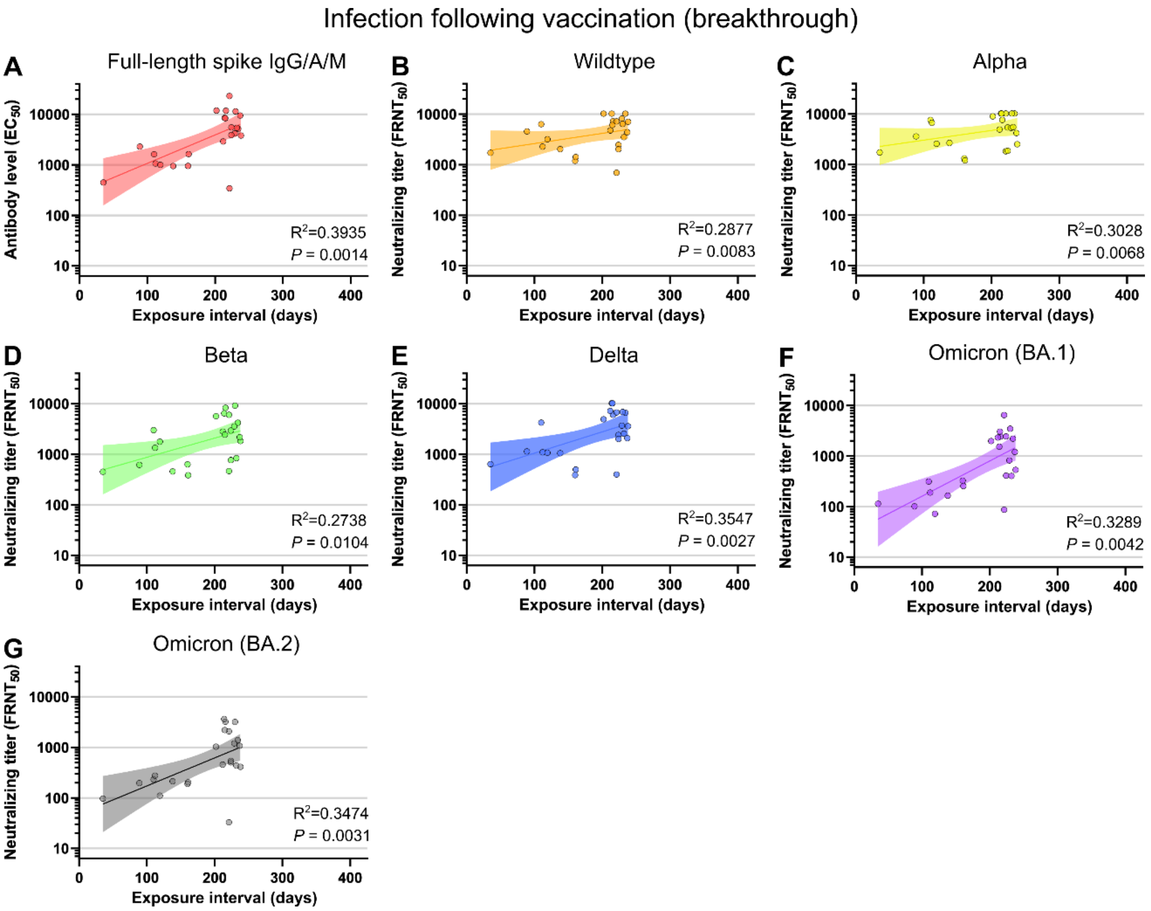

Supplemental figure 3: Vaccine breakthrough group neutralizing responses correlate with exposure interval

Comparison of exposure interval, the time between first and last antigen exposure, among individuals with vaccine breakthrough infections. Correlations are shown for full-length spike EC_{50} antibody levels (**A**), and neutralization of WA1 (**B**), Alpha (**C**), Beta (**D**), Delta (**E**), Omicron (BA.1) (**F**), and Omicron (BA.2) (**G**). Individual values are shown as filled circles and the shaded area indicates the linear fit with 95% confidence interval. R^2 is indicated for each curve fit and P values show the result of an F-test using a zero slope null hypothesis. All P values are two-tailed and 0.05 was considered significant. For panels A-G, n=23.

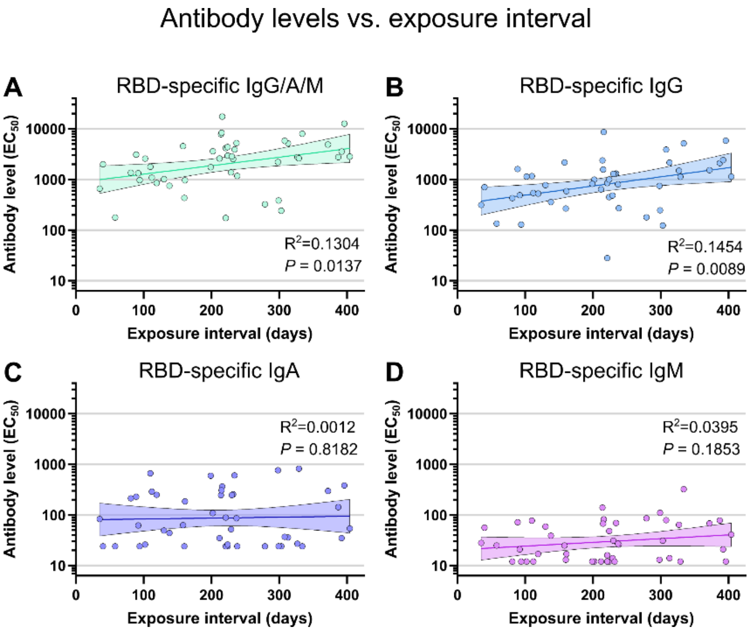

Supplemental figure 4: Other antibody isotypes correlate less well with exposure interval

Comparison of exposure interval, the time between first and last antigen exposure, with total (IgG/A/M) spike RBD (**A**), IgG (**B**), IgA (**C**), and IgM (**D**) EC_{50} antibody levels. Individual values are shown as filled circles and the shaded areas indicate the linear fit with 95% confidence interval. R^2 is indicated for each curve fit and P values show the result of an F-test using a zero slope null hypothesis. All P values are two-tailed and 0.05 was considered significant. For panels A-D, n=46.

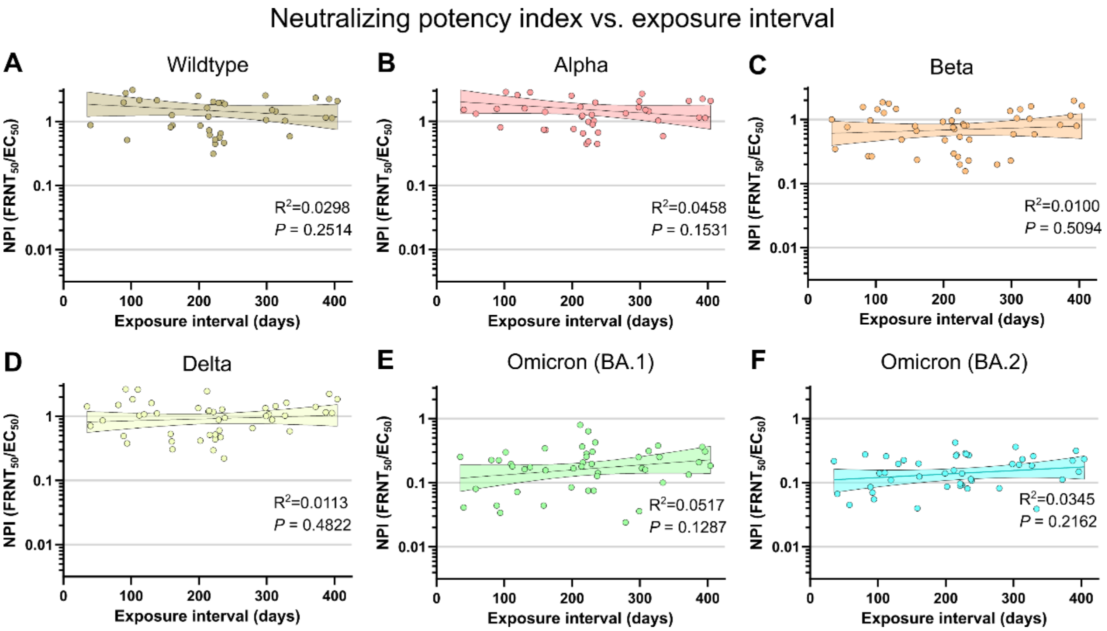

Supplemental figure 5: Neutralizing potency index does not correlate with exposure interval

Comparison of exposure interval, the time between first and last antigen exposure, with neutralization potency index ($FRNT_{50}$ / full-length spike EC_{50}) of wildtype (WA1) (**A**), Alpha (**B**), Beta (**C**), Delta (**D**), Omicron (BA.1) (**E**), and Omicron (BA.2) (**F**). Individual values are shown as filled circles and the shaded area indicates the linear fit with 95% confidence interval. R^2 is indicated for each curve fit. P values show the result of an F-test using a zero slope null hypothesis. All P values are two-tailed and 0.05 was considered significant. For panels A-F, n=46.
